# Characterization of extracellular polymeric matrix, and treatment of *Fusobacterium nucleatum* and *Porphyromonas gingivalis* biofilms with DNase I and proteinase K

**DOI:** 10.3402/jom.v5i0.20015

**Published:** 2013-01-29

**Authors:** Marwan Mansoor Ali Mohammed, Audun H. Nerland, Mohammed Al-Haroni, Vidar Bakken

**Affiliations:** 1 The Gade Institute – Section for Microbiology and Immunology, University of Bergen, Bergen, Norway; 2 Department of Pharmacy, Faculty of Health Sciences, University of Tromsø, Tromsø, Norway

**Keywords:** Subgingival biofilm, extracellular polymeric matrix, Fusobacterium nucleatum, Porphyromonas gingivalis, static and dynamic biofilm models, confocal laser scanning microscopy

## Abstract

**Background:**

Biofilms are organized communities of microorganisms embedded in a self-produced extracellular polymeric matrix (EPM), often with great phylogenetic variety. Bacteria in the subgingival biofilm are key factors that cause periodontal diseases; among these are the Gram-negative bacteria *Fusobacterium nucleatum* and *Porphyromonas gingivalis*. The objectives of this study were to characterize the major components of the EPM and to test the effect of deoxyribonuclease I (DNase I) and proteinase K.

**Methods:**

*F. nucleatum* and *P. gingivalis* bacterial cells were grown in dynamic and static biofilm models. The effects of DNase I and proteinase K enzymes on the major components of the EPM were tested during biofilm formation and on mature biofilm. Confocal laser scanning microscopy was used in observing biofilm structure.

**Results:**

Proteins and carbohydrates were the major components of the biofilm matrix, and extracellular DNA (eDNA) was also present. DNase I and proteinase K enzymes had little effect on biofilms in the conditions used. In the flow cell, *F. nucleatum* was able to grow in partially oxygenated conditions while *P. gingivalis* failed to form biofilm alone in similar conditions. *F. nucleatum* supported the growth of *P. gingivalis* when they were grown together as dual species biofilm.

**Conclusion:**

DNase I and proteinase K had little effect on the biofilm matrix in the conditions used. *F. nucleatum* formed biofilm easily and supported the growth of *P. gingivalis*, which preferred anaerobic conditions.

A biofilm has been defined as a structured community of bacterial cells enclosed in a self-produced extracellular polymeric matrix (EPM) and adherent to an inert or living surface (1). All biofilms share several common features – these include the production of extracellular polymeric substances (EPS), which are hydrated biopolymers secreted by bacteria. The biopolymers surround and immobilize microbial aggregates, make the macroscopic appearance of biofilms, and are frequently referred to as ‘slime’ (2).

In general, it is estimated that the microorganisms account for less than 10% of the dry weight of the biofilms, whereas the matrix can account for more than 90% (2). The EPM increases resistance to host defences and antimicrobial agents, compared with more vulnerable free-floating (planktonic) cells, and it forms a hydrated barrier between cells and their external environment. The function of the matrix includes adhesion, aggregation of microbial cells, cohesion of biofilm, retention of water, sorption of organic and inorganic material, enzymatic activity, nutrient source, exchange of genetic information, and export of cell components (2).

The EPM is chemically complex, varying with respect to bacterial species/strains and culture conditions (2, 3). Extracellular polysaccharides and proteins have been shown to be the key components of the matrix (2). Recent studies also indicate that extracellular DNA (eDNA) plays an important role in the establishment of biofilm structure (3–6). Some studies showed that removing eDNA reduces initial adhesion and aggregation of bacteria to surfaces (7, 8), and others have shown that eDNA is a major matrix component in some species biofilm (9), including *Pseudomonas aeruginosa* biofilm (10), where eDNA seems to induce antibiotic resistance (11). In *Staphylococcus aureus* biofilm, it was shown that cell lysis and the presence of eDNA were critical for attachment of biofilm during the initial stages of development and during biofilm maturation (12). Characterization of EPM components is mandatory in understanding biofilm structure and function. However, efficient EPM isolation is demanding because the isolation procedures might damage the cells causing contamination. Enzymatic treatment of biofilm was found to be helpful in the extraction of biofilm matrix, with no noticeable cell lysis (13, 14). Proteinase K is one of the enzymes that is used or included in the enzymatic extraction methods to degrade proteins in the matrix to increase nucleic acid release (14, 15).

Dispersal of biofilms by enzymes has been used in recalcitrant biofilms (e.g. using DNase I) on *P. aeruginosa* biofilms in cystic fibrosis patients (16). Treatment of biofilms with DNase I has also been shown to enhance the effect of antibiotics (17).

Oral bacterial biofilms are the key factors in the etiology of dental caries and periodontal diseases. The diversity, complexity, and multispecies community of the oral biofilm have been extensively reviewed (18, 19), but are still not fully clarified.

*Fusobacterium nucleatum* and *Porphyromonas gingivalis* are among the important species in the oral biofilm involved in the pathogenesis of periodontitis (20). *F. nucleatum* is commonly cultivated from the subgingival plaque from periodontitis patients, and because of its ability to aggregate with many oral bacteria, it works as a bridge between early and late colonizers in the dental biofilm (21). *P. gingivalis* is a member of the Socransky's red complex (bacteria strongly associated with periodontal disease) and has many virulent factors such as fimbriae, lipopolysaccharides, cysteine proteinases, and end products of metabolism (22).

The aim of this study was to characterize EPM main components and to analyse the effects of DNase I and proteinase K on early and mature biofilms formed by *F. nucleatum* and *P. gingivalis*. Confocal laser scanning microscopy (CLSM) was used in structural studies applying dynamic and static biofilm models.

## Materials and methods

### Bacteria and growth medium

*Fusobacterium nucleatum* subsp. *nucleatum*, strain ATCC 25586 and *P. gingivalis* type strains ATCC 53978 (W50), ATCC 33277, and ATCC BAA-1703 (FDC 381) were used in the current study.

The bacteria were grown on fastidious anaerobic agar (FAA) plates at 37°C in anaerobic condition (5% CO_2_, 10% H_2_, and 85% N_2_) (Anoxomat System, MART Microbiology, Lichtenvoorde, The Netherlands) for 48 h and then inoculated in liquid medium prepared with the following: tryptone (Oxoid Ltd., London) (15 g/L); NaCl (5 g/L); KH_2_PO_4_ (1.5 g/L); Na_2_HPO_4_·2H_2_O (3.5 g/L); NaHCO_3_ (0.5 g/L); and yeast extract (Oxoid) (3.0 g/L). Filter sterilized ascorbic acid (1 mg/L), vitamin B12 (0.1 mg/L), glucose (5.5 g/L), and hemin (5 mg/L) were added to the autoclaved part of the medium (23). The bacteria were incubated for 24 h at 37°C in anaerobic conditions and were used as the source of culture inoculum in the dynamic and static biofilm models (see beneath).

### The flow cell biofilm

Biofilms were grown at 37°C in three-channel flow cells with individual channel dimensions of 1×4×40 mm. The flow system was assembled and prepared as described by Christensen et al. (24). A glass cover slip (24×50 mm) (product # 1014; Assistant, Sondheim/Rhön, Germany) was used as substratum for biofilm growth. Before each experiment was carried out, the flow cell system was autoclaved, and after assembling, the system was sterilized by pumping a 0.5% (wt/vol) hypochlorite solution into the system and leaving it there for 4 h. The system was flushed with 2 L of sterile water. The flow chamber was then filled overnight with media at 37°C to let the system equilibrate with the medium. Inocula were prepared as follows: bacteria grown for 48 h on FAA plates were re-suspended in liquid media and incubated overnight at 37°C. After adjusting the optical density at 550 nm to 0.5, aliquots of 250 µl cultures were injected into each channel of the flow cell after stopping the medium flow and clamping off the silicon tubing to prevent back flow into the system. The flow cell was inverted for 1 h to allow for adhesion of cells to the glass surface without flow. The flow was then resumed and the clamps were removed. During the growth of biofilms, the fresh medium was pumped through the flow cells at a constant rate of 3.3 ml/h/channel by using a peristaltic pump (model 205S; Watson-Marlow, Falmouth, UK) (25).

### The biofilm for EPS extraction

Petri dishes with 9 cm diameter (Nunc, Rochester, NY, USA) each containing 20 ml of liquid medium were inoculated with 100 µl of bacterial suspension (OD_550nm_=1). The dishes were incubated in anaerobic conditions (without shaking) at 37°C for 5 days. The medium was then removed and the biofilm samples were washed twice with phosphate-buffered saline (PBS) before being harvested by scraping with a cell scraper (Nunc, Rochester, NY, USA). The biofilm samples were suspended in 1 ml PBS and stored at −20°C until processing.

### Enzymatic treatment of harvested biofilm

The biofilm samples were homogenized with FastPrep FP120 Thermo Savant homogenizer (Qbiogene, Cedex, France) at a speed of 4 m/sec for 20 sec, then Proteinase K was added to 500 µl of each sample to a final concentration of 5 µg/ml as described (14, 26). Samples with added distilled water were used as controls. Enzyme treated samples and controls were incubated at 37°C for 1 h. After enzymatic treatment, the biofilm samples and controls were filtered through 0.2 µm pore size acrodisc syringe filters (Pall, BioSciences, Ann Arbor, MI, USA). Aliquots from the eluate were used for quantification of proteins and carbohydrates and extraction of DNA.

### Protein concentration assay

For measurement of the protein concentration, the samples and controls were diluted 10 times in distilled water, and then 0.5 ml of Lowry reagent was added to 0.5 ml of sample dilution. After 20 min at room temperature, 0.5 ml of Folin and Ciocalteu's phenol reagent working solution (Sigma-Aldrich, MO, USA) were added to the mixture and left for another 30 min at room temperature (25). The absorbance of the standards and samples were measured at 750 nm and compared to a standard curve obtained by serial dilution of bovine serum albumin.

### Carbohydrate assay

The carbohydrate concentration in EPM was measured by the anthrone method with the modifications described by Raunkjær et al. (14, 27), using glucose as a reference standard. The samples and controls were prepared by 10 times dilution in distilled water, and then 100 µl of each dilution was mixed with 200 µl of anthrone reagent (0.125% anthrone [wt/vol] in 94.5% [vol/vol] H_2_SO_4_). Samples and controls were placed in a water bath at 100°C for 14 min and then cooled at 4°C for 5 min. The absorbance at 595 nm was measured using microtitre plate reader (Multiskan MS Type 352, Labsystems, Finland).

### eDNA extraction and quantification

Extraction of eDNA was performed using Fast DNA spin kit (MP Biomedicals, Solon, OH, USA) according to the manufacturer's instructions. Measurements of DNA concentration in 500 µl from each sample were carried out using a NanoDrop spectrophotometer (Thermo Fisher Scientific Inc. Waltham, MA, USA).

The eDNA was electrophoresed on an 0.8% agarose gel from SeaKem (FMC BioProducts, Rockland, ME, USA) and stained with GelRed™ (Biotium, Hayward, CA, USA) using 0.5×TBE buffer at 100 V for 40 min. EZ load 100-bp molecular ruler (Bio-Rad, CA, USA) was used as DNA standard.

### Static biofilm microtitre plate assay

Ninety-six Well Black with Clear Flat Bottom Polystyrene Not Treated Microplates (cat. no. 3631, Corning, NY, USA) were used to grow biofilms. The effect of the enzymes was evaluated on biofilm formation and mature biofilm (26). Deoxyribonuclease I (DNase I) (Sigma-Aldrich, MO, USA) from bovine pancreas was prepared in an enzyme buffer (0.15 mM NaCl and 5 mM MgCl_2_), and proteinase K (Sigma-Aldrich, MO, USA) was prepared in distilled water. The two enzymes were used in different concentrations (0.125, 0.25, 0.5, and 1 mg/ml). The enzyme buffer (for DNase I) and distilled water (for proteinase K) were used for the controls. The bacteria were prepared by diluting overnight grown bacterial cultures to prepare suspensions of 1.2×10^7^ cfu/ml.

A total of 200 µl from the bacterial suspension was used in each well of the microplates to grow biofilm. For dual species biofilm, equal amounts (100 µl) from each bacterium were used.

To evaluate the effect of DNase I and proteinase K on biofilm formation, the enzymes were added and then the microplates were incubated in anaerobic conditions at 37°C for 48 h. To evaluate the effects on mature biofilm, a 48-h-old biofilm was washed with PBS, and then the enzymes were added in their respective buffers and incubated for 1 h at 37°C.

The medium and enzymes were removed, and the wells were washed once with distilled water. The biofilm was then stained with 150 µl of crystal violet (0.5%) for 15 min, the stain was removed, and the biofilm was washed twice with distilled water and left to dry. To solubilize the stain, 150 µl of 95% ethanol was added to each well, and the absorbance was read at 570 nm in an automatic ELISA microplate reader (Multiskan MS Type 352, Labsystems, Finland).

CLSM was used to visualize the effect of enzymes on biofilm formation and on mature biofilm. In brief, the biofilm was grown in *µ*-clear bottom, chimney well, surface treated, sterile 96 Well Microtitre plates (cat. no. 635090, Greiner Bio-One, Frickenhausen, Germany) under the same conditions as described above. The concentrations of DNase I and proteinase K used on the biofilm examined by CLSM were 1 mg/ml.

### CLSM of biofilms in flow cells and microtitre plates

The biofilms were examined by Zeiss LSM 510 META equipped with a water-immersion of 63×objective (Carl Zeiss, Jena, Germany). The biofilms were stained for 15 min with 100 µl LIVE/DEAD BacLight Bacterial Viability Kit (Invitrogen Corporation, NY, USA). The final concentrations of Syto-9 and propidium iodide (PI) were 0.01 mM and 0.06 mM, respectively. The SYPRO^®^ Ruby biofilm matrix stain (Invitrogen Corporation, NY, USA) was used to stain proteins in the EPM. The green fluorescence and red fluorescence of SYTO 9 and PI were excited using an argon laser beam, with excitation lines at 488 nm and a helium/neon at 543 nm, respectively. The SYPRO^®^ Ruby stain was excited at 405 nm with diode laser. The CLSM image stacks were analysed by the image-processing software COMSTAT (28). The biomass, average thickness, and maximum thickness were the parameters used to compare different biofilms.

### Statistical analyses

The software package IBM SPSS 19.0 was used for the statistical analyses. The means and standard deviations of carbohydrates and eDNA concentrations in harvested biofilms that had been treated with proteinase K were calculated, and Mann–Whitney U-test was used to compare the means. The means and standard deviation of absorbance values representing the effect of DNase I and proteinase K on early biofilm formation or mature biofilm were calculated for each enzyme concentration and each tested biofilm. Multiple comparisons within groups were performed by the Kruskal–Wallis test, and, if significant, the Mann–Whitney U-test was used as a post hoc test. The significance level was set to *p*<0.05.

## Results

### Biofilm growth in flow cell

**Fig. 1:**
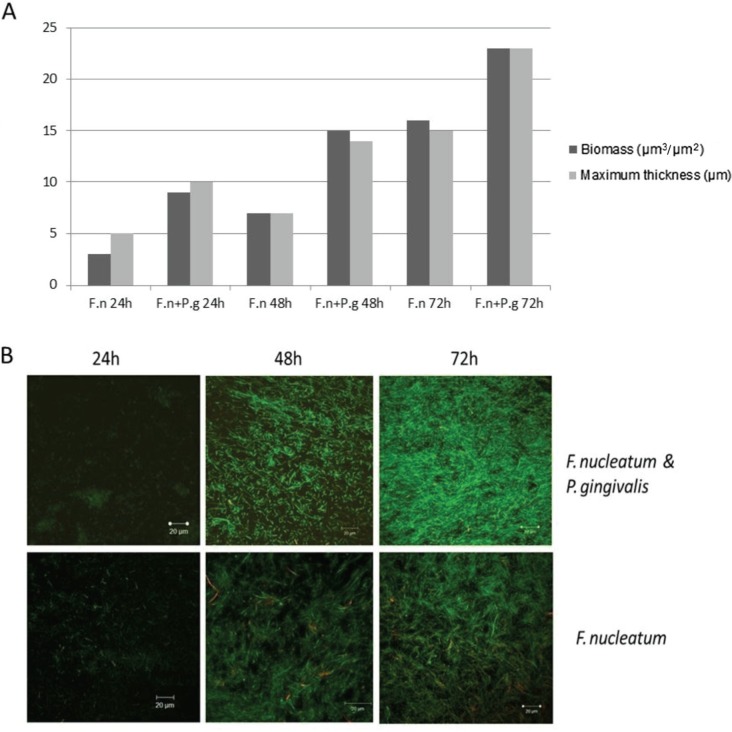
(A) Biomass and maximum thickness of *Fusobacterium nucleatum* ATCC 25586 (F.n) when grown alone or together with *Porphyromonas gingivalis* W50 (P.g) biofilm and calculated from one point z-stack confocal microscopy images taken from the middle of the flow cell and analysed by COMSTAT software program. (B) Representative CLSM images of 24-h- (left), 48-h- (middle), and 72-h- (right) old biofilms showing mutualistic growth of *F. nucleatum* ATCC 25586 and *P. gingivalis* W50 (upper) compared to mono-species *F. nucleatum* ATCC 25586 (lower). The biofilms were grown in flow cells and were stained with Cyto9 (green) and propidium iodide (red).

*F. nucleatum* was able to form biofilm in partially oxygenated conditions where the biofilm developed shortly (2–4 h) after inoculation. *P. gingivalis* failed to form biofilm alone in similar conditions. However, the growth of *P. gingivalis* was initiated when grown together with *F. nucleatum*. Biofilm formation and maturation were enhanced by co-culture of the two species (Fig. 1A and B).

**Fig. 2:**
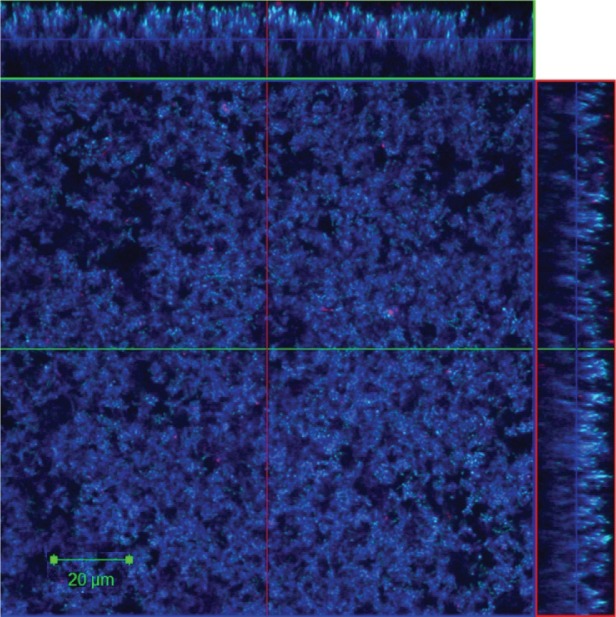
Representative CLSM image shows proteins in EPM of 24-h dual species biofilm (*Fusobacterium nucleatum* ATCC 25586 and *Porphyromonas gingivalis* W50) grown in flow cells. The proteins were stained with SYPRO^®^ Ruby stain (blue). Sideviews, XZ (top) and YZ (right) are sagittal sections of the biofilm. Scale Bar = 20 µm.

The CLSM images revealed irregular topography of the biofilm, without clear mushroom-shaped structure (Figs. 1B and 2). The biofilm thickness after 3 days of cultivation ranged from 20–30 µm in our experimental setting.

### The EPM major components

The EPM components were extracted from the static biofilm. Proteins and carbohydrates were major components of the biofilm matrix, and the protein concentration in the samples of the extracellular biofilm ranged from 374 to 982 µg/ml, with an average of 666 µg/ml, and for carbohydrate, the concentration ranged from 348 µg/ml to 990 µg/ml, with an average of 682 µg/ml. For DNA, the concentration ranged from 17 to 46 µg/ml, with an average of 25 µg/ml.

**Fig. 3:**
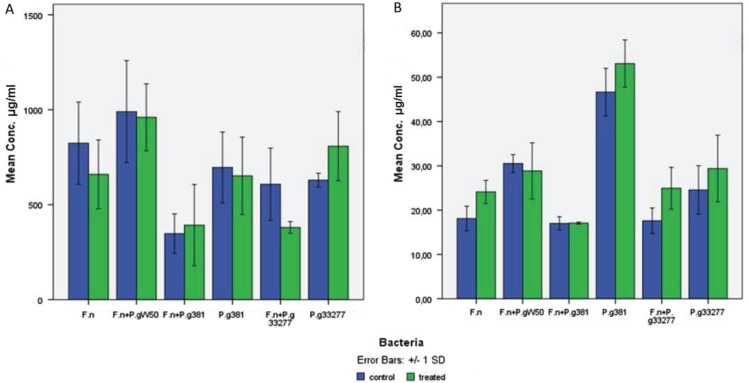
Comparison of (A) carbohydrate and (B) eDNA yields from the biofilm matrix samples treated with proteinase K enzyme and non-treated samples. The matrix of 5-day-old biofilm was treated with 5 µg/ml proteinase K at 37°C for 1 h. F.n, *Fusobacterium nucleatum* ATCC 2558; P.g W50, *Porphyromonas gingivalis* W50; P.g 381, *P. gingivalis* FDC381; P.g 33277, *P. gingivalis* ATCC 33277. The bars represent the means with standard deviations from five samples (carbohydrates) and three replicates (eDNA).

Chemical analysis of EPM showed that the contents of proteins and carbohydrates were highest in EPM extracted from *F. nucleatum* and *P. gingivalis* W50 biofilm and lowest in *F. nucleatum* and *P. gingivalis* FDC381 biofilm (Fig. 3).

Proteins in the EPM of the dual species *F. nucleatum* and *P. gingivalis* biofilm grown in flow cells were visualized by CLSM after staining with Live/Dead and SYPRO^®^ Ruby biofilm matrix stain, and it showed abundant amounts of proteins distributed within the biofilm matrix (Fig. 2).

**Fig. 4:**
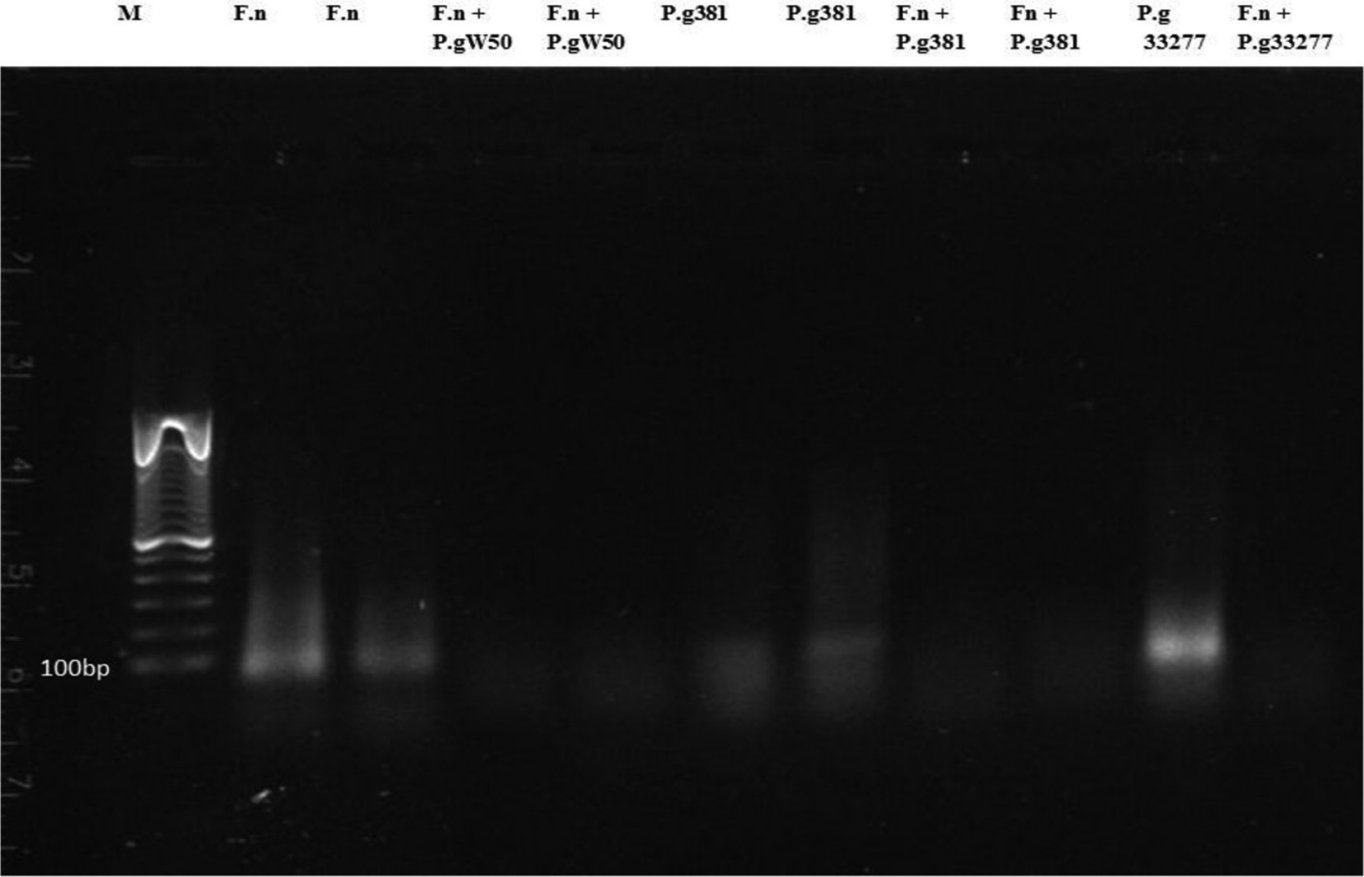
Gel electrophoresis (0.8% agarose gel) of the extracellular DNA. The eDNA was extracted from the matrix of 5-day-old biofilm of these species. F.n, *Fusobacterium nucleatum* ATCC 25586; P.g W50, *Porphyromonas gingivalis* W50; P.g 381, *P. gingivalis* FDC381; P.g 33277, *P. gingivalis* ATCC 33277. Lane M, EZ load 100-bp molecular ruler (Bio-Rad).

No statistical significant differences were found in the concentrations of carbohydrates and eDNA between Proteinase K-treated and non-treated harvested biofilms (Fig. 3). The extracted DNA was analysed by agarose gel electrophoresis and the size of eDNA was found to be around 100 bp as shown (Fig. 4).

### Enzyme effect on biofilm formation and mature biofilm

**Fig. 5:**
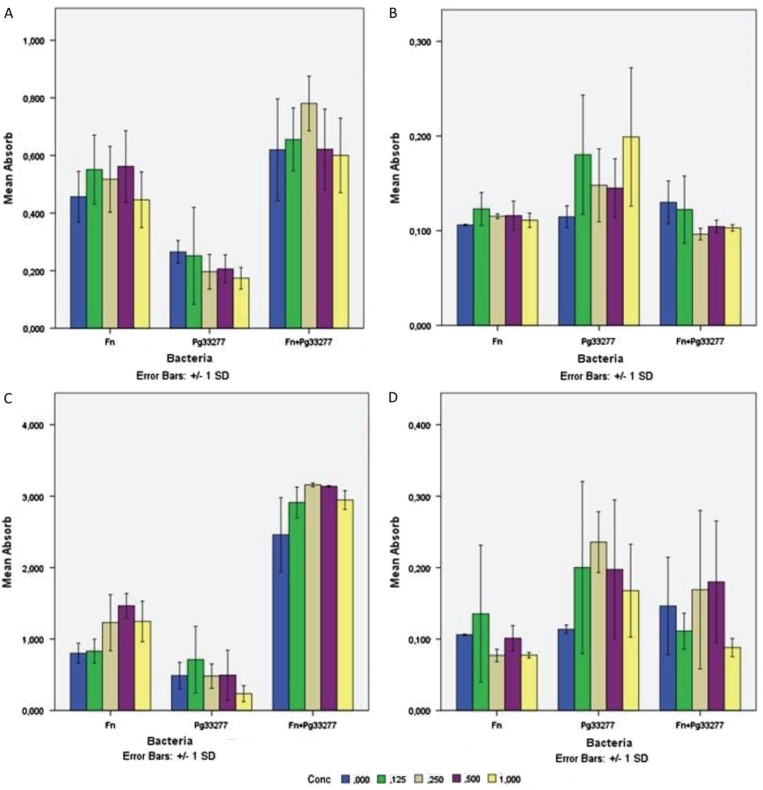
Effect of DNase I and proteinase K on the biofilm formation (time zero) and on 48-h-old biofilm. *Fusobacterium nucleatum* type strain ATCC 25586 (F.n) and *Porphyromonas gingivalis* type strain ATCC 33277 (P.g 33277) bacterial species were tested when they were grown as monoculture or as dual species culture. (A) DNase I effect on biofilm formation, (B) DNase I effect on 48-h biofilm, (C) Proteinase K effect on biofilm formation, (D) Proteinase K effect on 48-h biofilm. The colored columns refer to the enzyme concentrations (0.125, 0.25, 0.5, and 1 mg/ml). The y-axis represents absorbance at 570 nm. The bars represent the means with standard deviations for 3–5 samples.

To test the effect of DNase I and proteinase K on biofilm formation, the enzymes were added at time zero, and the biofilm was analysed after 48 h. To test the effect on mature biofilm, the enzymes were added at 48 h, and the effect was analysed after 1 h of incubation. *F. nucleatum* type strain ATCC 25586 and *P. gingivalis* type strain ATCC 33277 were tested when they were grown as a monoculture or as a dual species culture. In the static biofilm model (microtitre plates), the effects of these enzymes were not statistically significant (Fig. 5).

**Fig. 6:**
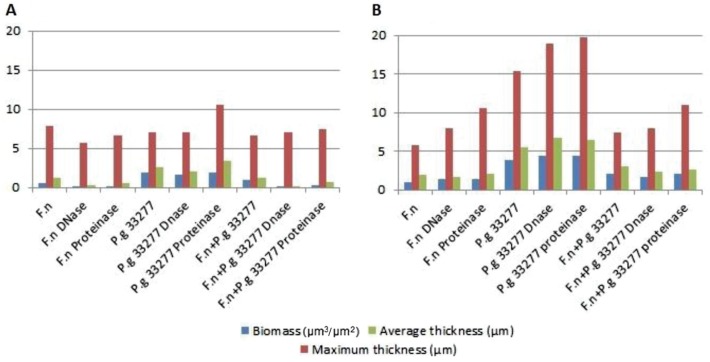
Effect of DNase I and proteinase K enzymes at a concentration of 1 mg/ml on biofilm formation, (A) time zero and (B) 48-h-old biofilm. The graphs show the biomass (µm^3^/µm^2^), maximum thickness (µm), and average thickness (µm) of one point z-stack confocal images analysed by COMSTAT software program. F.n, *Fusobacterium nucleatum* ATCC 25586; P.g 33277, *Porphyromonas gingivalis* ATCC 33277.

These findings were confirmed by CLSM image analyses, where bacterial biomass, maximum thickness, and average thickness of the biofilm were measured (Fig. 6). These parameters have little or no variation after enzymatic treatment, and the biofilm shape and structure remained unchanged.

## Discussion

There is a lack of studies on EPM of subgingival biofilm. However, exo-polysaccharides in the supragingival biofilm have been extensively studied and known for a long time (29–31). In the present study, we have isolated EPM and tested effects of DNase I and proteinase K on biofilms from periodontal-disease-associated bacteria grown *in vitro*.

The EPM of the bacterial biofilms in our study are characterized by the presence of macromolecular complexes of carbohydrates, proteins, and nucleic acids. The enzymatic treatment of the harvested biofilms with proteinase K was performed to find out if it would increase the liberation of eDNA or carbohydrates, compared with only vortexing or homogenizing. This treatment did not result in noticeable difference in the yielded eDNA or carbohydrates (Fig. 3). Wu and Xi found similar results for carbohydrates in *Acinetobacter* sp. strain AC811 biofilm matrix, but the eDNA yield was increased after enzyme treatment (14).

Even though proteins were abundant in the biofilm matrix of our *F. nucleatum* and *P. gingivalis* dual species biofilm as shown in (Fig. 2), treatment with proteinase K was shown to be insufficient to disperse the biofilm matrix. A carbohydrate-rich matrix might be the reason for this ineffectiveness, which has also been suggested for staphylococcal biofilms (32, 33). The eDNA detected in the EPM in our biofilms had a size around 100 bp, as demonstrated with agarose gel electrophoresis (Fig. 4). This size is usually higher than described for other biofilms; however, the size of the eDNA has been reported to range from less than 100 bp to 10 kb (34). Targeting the eDNA with DNase I in the mono or dual species biofilm matrix in our study gave no obvious effects with respect to prevention of biofilm formation or dispersion of mature biofilm. This is in contrast to enzymatic treatment of *P. aeruginosa* biofilm (4). One suggested function of eDNA is in gene transfer (35, 36). The biofilm may offer an excellent milieu for DNA exchange, as the cells are in close juxtaposition and DNA can be trapped within the extracellular matrix (37). Genus *Fusobacterium* and other genera of oral bacteria contain conjugative transposons that facilitate the DNA transfer between bacteria through conjugation. *P. gingivalis* also shows a large degree of variation between strains, proposing that this organism has gone through frequent genetic rearrangements (37, 38).

In this study, we have shown that *F. nucleatum* can grow in a flow cell biofilm model in a non-strictly anaerobic environment, while this was not true for *P. gingivalis*. It seems to be a synergistic enhancement in the biofilm formation when these two species are grown together even in a partially oxygenated condition, which indicates that *F. nucleatum* might have the capacity to protect *P. gingivalis* from oxidative stress. This has also been reported in other studies (39, 40). Nearby *in vivo* association between these two microorganisms might indicate that they support each other, as shown in biofilm and mouse models (41, 42). *F. nucleatum* and *P. gingivalis* have been found to co-aggregate *in vitro* and *in vivo*, which could play a role in biofilm formation and pathogenesis, as also reported from mouse model experiments (43).

In general, a better understanding of the EPM of subgingival biofilm and complex multispecies biofilms should lead to more efficient control strategies. The management of biofilm growth does not necessarily require direct killing of the bacteria in the biofilm, but it might be directed toward degradation or dispersal of the biofilm matrix to reverse the biofilm mode of growth to a planktonic state, which is significantly easier to treat and manage, for example, by antibiotics.

In conclusion, in this study, we demonstrate that proteins and carbohydrates are major components in the EPM biofilms of *F. nucleatum* and *P. gingivalis* grown *in vitro*; however, eDNA might also play a role in the structuring of the biofilm. More detailed structural and functional studies of EPM of subgingival biofilms are needed to identify and to attack new targets to control biofilm growth. Improved models and more complex systems and *in vivo* studies are clear objectives for further work.
